# Deep learning applied to standard radiographs improves detection of implant loosening in total knee arthroplasty: A proof‐of‐concept study

**DOI:** 10.1002/jeo2.70611

**Published:** 2026-01-19

**Authors:** Kelly Mills, Guido A. de Jong, Simon N. van Laarhoven, Thomas J. J. Maal, Petra J. C. Heesterbeek

**Affiliations:** ^1^ Research Department Sint Maartenskliniek Nijmegen the Netherlands; ^2^ Department of Medical Imaging, 3D Lab Radboud UMC Nijmegen the Netherlands; ^3^ Orthopaedics Department Sint Maartenskliniek Nijmegen the Netherlands

**Keywords:** artificial intelligence, aseptic loosening, convolutional neural networks, deep learning, total knee arthroplasty

## Abstract

**Purpose:**

Implant loosening is a common cause of failure in total knee arthroplasty, and for appropriate treatment, the correct diagnosis is of vital importance. Detection is challenging as radiographs only reveal obvious cases, computed tomography (CT) scans are hindered by metal artifacts, and bone scintigraphy lacks specificity, while all methods are time‐consuming and/or involve radiation exposure. This proof‐of‐concept study aimed to develop a deep learning (DL) tool to detect implant loosening using a set of two‐dimensional (2D) radiographs.

**Methods:**

A total of 307 radiograph sets (anteroposterior and lateral) were collected, including 159 loose and 148 fixed primary knee implants (confirmed intraoperatively during revision surgery). Images were square‐cropped, centred on the implant, horizontally flipped for left knees, resized to 512 × 512 pixels and standardized for grayscale. A dual InceptionV3 DL algorithm was trained using fivefold cross‐validation. Performance was assessed using sensitivity, specificity, accuracy and receiver operating characteristic curves, with corresponding area under the curve (AUC) and compared to routine radiological reports.

**Results:**

The mean ± standard deviation (SD) sensitivity, specificity and accuracy over the fivefolds were 71.7% ± 10.7%, 87.0% ± 14.2% and 79.2% ± 4.0%, respectively. The overall AUC was 0.81, confidence interval (CI): [0.77–0.85]. The radiological report predictions reached a sensitivity of 51.3%, specificity of 99.2% and accuracy of 73.2%. The algorithm predicted significantly more cases correctly compared to standard radiological evaluations by a musculoskeletal specialized radiologist (*p* = 0.005).

**Conclusions:**

This proof‐of‐concept study demonstrated the feasibility of training a deep‐learning algorithm using 2D radiographs to detect implant loosening. Notably, the algorithm outperformed routine radiological evaluations, highlighting its potential to enhance the detection of loosening. These results are promising, considering the limited dataset. This tool could be valuable for patients experiencing issues after knee replacement surgery, helping to rule out implant loosening while reducing analysis time, radiation exposure and healthcare costs.

**Level of Evidence:**

Level III.

## Graphical abstract

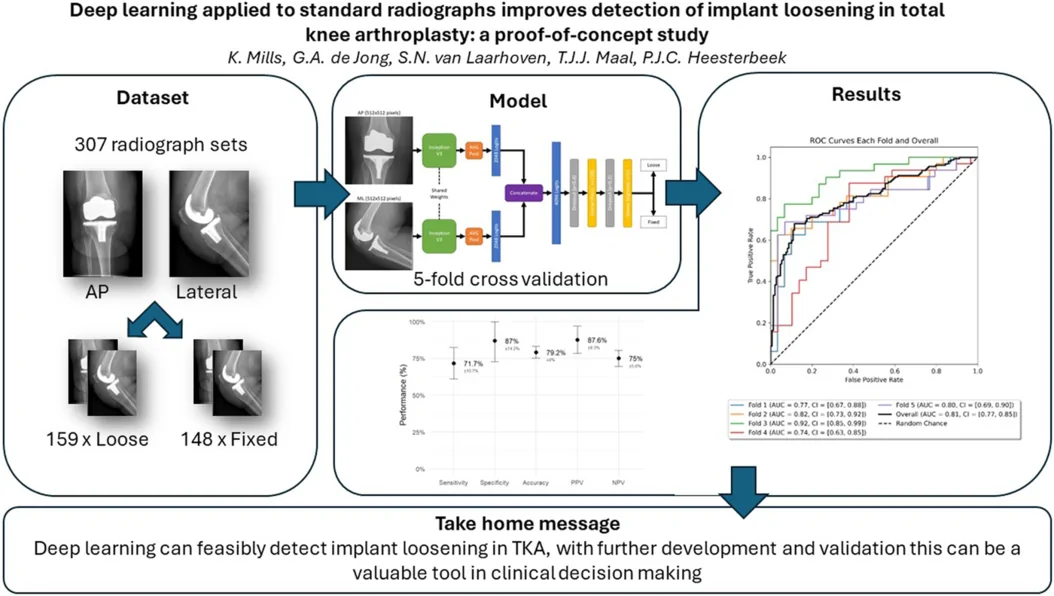

**Abbreviations**
2D: two‐dimensional
AI: artificial intelligence
AP: anteroposterior
AUC: area under the curve
AVG: average
CI: confidence interval
CLAIM: Checklist for Artificial Intelligence in Medical Imaging
CR: computed radiography
CT: computed tomography
DL: deep learning
DX: digital radiography
ML: mediolateral
NPV: negative predictive value
PPV: positive predictive value
PS: posterior stabilized
ROC: receiver operating characteristics
rTKA: revision total knee arthroplasty
SD: standard deviation
THA: total hip arthroplasty
TKA: total knee arthroplasty

## INTRODUCTION

Implant loosening is a leading cause of revision in total knee arthroplasty (TKA) [1], accounting for over 20% of all revision procedures [5, 17]. Revision surgery is technically demanding, costly and associated with higher complication rates and poorer outcomes compared to primary arthroplasty [8]. Although overall revision rates remain relatively low, the increasing number and younger age of TKA recipients are expected to impose a growing clinical and economic burden [13]. Performing revision surgery without clear evidence of loosening often leads to suboptimal outcomes [3]. Conversely, retaining a loose implant can exacerbate symptoms, increase bone stress, and lead to bone loss and reduced bone quality [7], ultimately complicating subsequent revision procedures.

Currently diagnostic assessment of implant loosening relies primarily on visual evaluation of the fixation interface between implant and bone. Standard two‐dimensional (2D) radiographs are useful when loosening is advanced and radiolucency, osteolysis, or migration are visible, but early changes often go undetected. Computed tomography (CT) provides a three‐dimensional view, but metal artifacts often obstruct clear visualization of the interface, and interpretation still relies on the radiologist's visual and subjective assessment. In addition, CT exposes patients to higher radiation doses and requires substantial review time from medical personnel. Bone scintigraphy can also assist in diagnosing loosening [11], but its specificity is limited, as it does not reliably differentiate loosening from infection or other causes of increased bone turnover. Moreover, it is time‐consuming and costly.

Artificial intelligence (AI), particularly deep learning (DL) approaches, offers a potential solution to these limitations [4]. DL models can detect subtle imaging patterns that are difficult to detect for human observers. Thereby, they could improve detection accuracy and reduce its subjectivity and workload.

The primary aim of this proof‐of‐concept study was to develop a prototype DL‐based tool to detect implant loosening using retrospective clinical and radiological data from confirmed loose and fixed cases. It was hypothesized that this tool would be able to distinguish loose from fixed implants with performance exceeding chance level, thereby demonstrating the feasibility of this approach for future clinical application.

## METHODS

For this proof‐of‐concept study, 307 radiographic sets (mediolateral [ML] and standing anteroposterior [AP] knee views), with corresponding clinical data on implant fixation status, were extracted from the Sint Maartenskliniek's electronic patient records. These included 159 loose and 148 fixed implants. Loose TKAs were defined as those showing radiological suspicion of loosening on radiograph, CT or bone scintigraphy, combined with intraoperative confirmation of loosening during revision TKA (rTKA). Fixed implants were defined as cases indicated for minor revision procedures (liner exchange or secondary patellar resurfacing) without radiological evidence of loosening and without signs of mechanical loosening during rTKA. The dataset included three of the most frequently used implant types in this clinic: the Genesis II posterior stabilized (PS) (Smith and Nephew), NexGen PS (Zimmer Biomet) and Vanguard PS (Zimmer Biomet). Patients with confirmed periprosthetic infection or revision for malalignment were excluded. Radiographic images were captured either at the Sint Maartenskliniek or imported following referral from other clinics, between October 2012 and November 2023. All images were captured using Philips DigitalDiagnost (*n* = 246), Canon Inc. CXDI (*n* = 45), Siemens Fluorospot Compact FD (*n* = 13), or Samsung Electronics GC85A (*n* = 2), with one image from an unknown system. Of the total dataset, 66 radiograph sets were obtained using computed radiography (CR), whereas 241 sets were acquired using digital radiography (DX). The median maximum high voltage applied across the radiographs was 66 kV (range: 54.9–74.0 kV). The study was conducted in accordance with relevant guidelines and regulations and is reported following the Checklist for Artificial Intelligence in Medical Imaging (CLAIM) guidelines [20]. Informed consent was waived because only retrospective anonymized data were used.

### Image preprocessing

A custom Python‐based graphical user interface was used to manually square‐crop all images, centred on the midpoint of the implant, while ensuring that the surrounding tibial bone was fully included within the crop. All images of left knees were subsequently horizontally flipped (*n* = 141) to match the orientation of right knees. After this, all images were resized to 512 × 512 pixels and standardized to grayscale by subtracting the mean pixel intensity and dividing by the standard deviation. During model training, data augmentation techniques were applied, including random horizontal and vertical flipping and intensity shifts ranging from −10% (0.9) to +10% (1.1). Training images were processed in batches of 8, while test images were processed individually (batch size = 1). Paired radiographs (ML and AP views) were used as input for the DL algorithm.

## DEEP LEARNING

**Figure 1:**
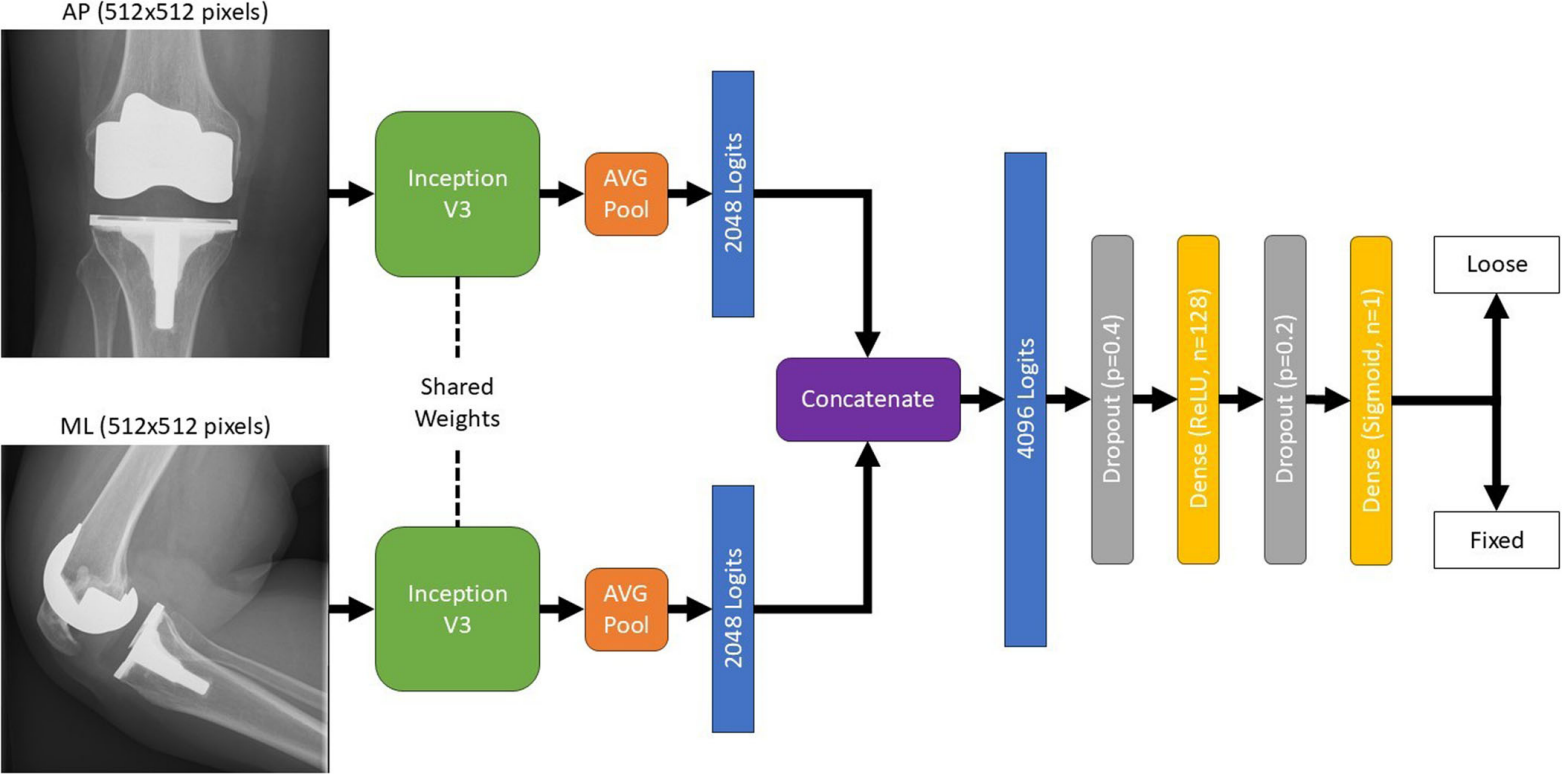
Schematic overview of the deep learning network architecture. AP, anteroposterior; AVG, average; ML, mediolateral.

The study employed a parallel InceptionV3 network architecture with shared weights as illustrated in Figure 1. The model concatenated the average pooled latent outputs (two sets of 2048 values) into a single 4096‐dimensional layer, followed by:

1. A dropout layer (rate = 0.4) to prevent overfitting.
2. A fully connected (dense) layer with 128 neurons and Rectified Linear Unit (ReLU) activation.
3. Another dropout layer (rate = 0.2).
4. A final dense layer with a single neuron and Sigmoid activation for classification.

Both InceptionV3 networks (one for ML, one for AP) shared weights, ensuring simultaneous updates during training. The model was optimized using Adam (learning rate = 1E‐5) with binary cross‐entropy loss. Training was conducted on TensorFlow 2.10 using an NVIDIA Titan V (12GB VRAM).

A fivefold cross‐validation strategy was used, with a 4:1 training‐to‐test split. Performance metrics were reported for each test fold as well as for the overall dataset. The algorithm and trained model used in this study are available from the corresponding author upon reasonable request.

### Statistics

Receiver operating characteristic (ROC) curves, and the corresponding area under the curve (AUC), were calculated for each fold separately, as well as for the entire dataset. The 95% confidence interval (CI) for the AUC was estimated using DeLong's method.

To identify optimal cut‐off values, Youden's index statistic was computed (*J* = Sensitivity + Specificity − 1) across 100 evenly spaced cut‐off values, ranging from the minimum to the maximum prediction value. The cut‐off value maximizing Youden's index was selected, and predictions were generated for each fold. Algorithm performance for each fold was summarized in a confusion matrix.

Data were analysed to ensure even distribution of implant types across folds and a balanced distribution of fixation status (loose vs. fixed) within each implant group. Equal distribution between groups was verified using chi‐square tests. Algorithm performance was evaluated separately for each implant type. Pairwise comparisons of sensitivity, specificity, accuracy, positive predictive value (PPV), and negative predictive value (NPV) were performed using *z* tests for independent proportions.

Routine radiological reports were reviewed to classify each implant as either loose or fixed, based on the assessment of an experienced musculoskeletal radiologist. If fixation status was not reported, or if no report was available, the data were marked as missing. Cases in which radiolucencies or possible loosening were reported, but without clear confirmation of loosening, were classified as loose. Radiological report predictions were compared to the algorithm's predictions using McNemar's test [16]. A significance level of 0.05 was applied. All statistical analyses were performed using Stata version 17.0 (StataCorp LCC).

Gradient‐weighted Class Activation Mapping (Grad‐CAM) was used to visualize which regions in the radiographs contributed most to the model's prediction. Grad‐CAM heatmaps were generated from the last convolutional layers of the InceptionV3 backbone using the standard gradient‐based approach. Because both the AP and lateral images were processed through a shared convolutional backbone, the resulting activation maps reflect shared feature representations rather than strictly projection‐specific attention.

## RESULTS

**Figure 2:**
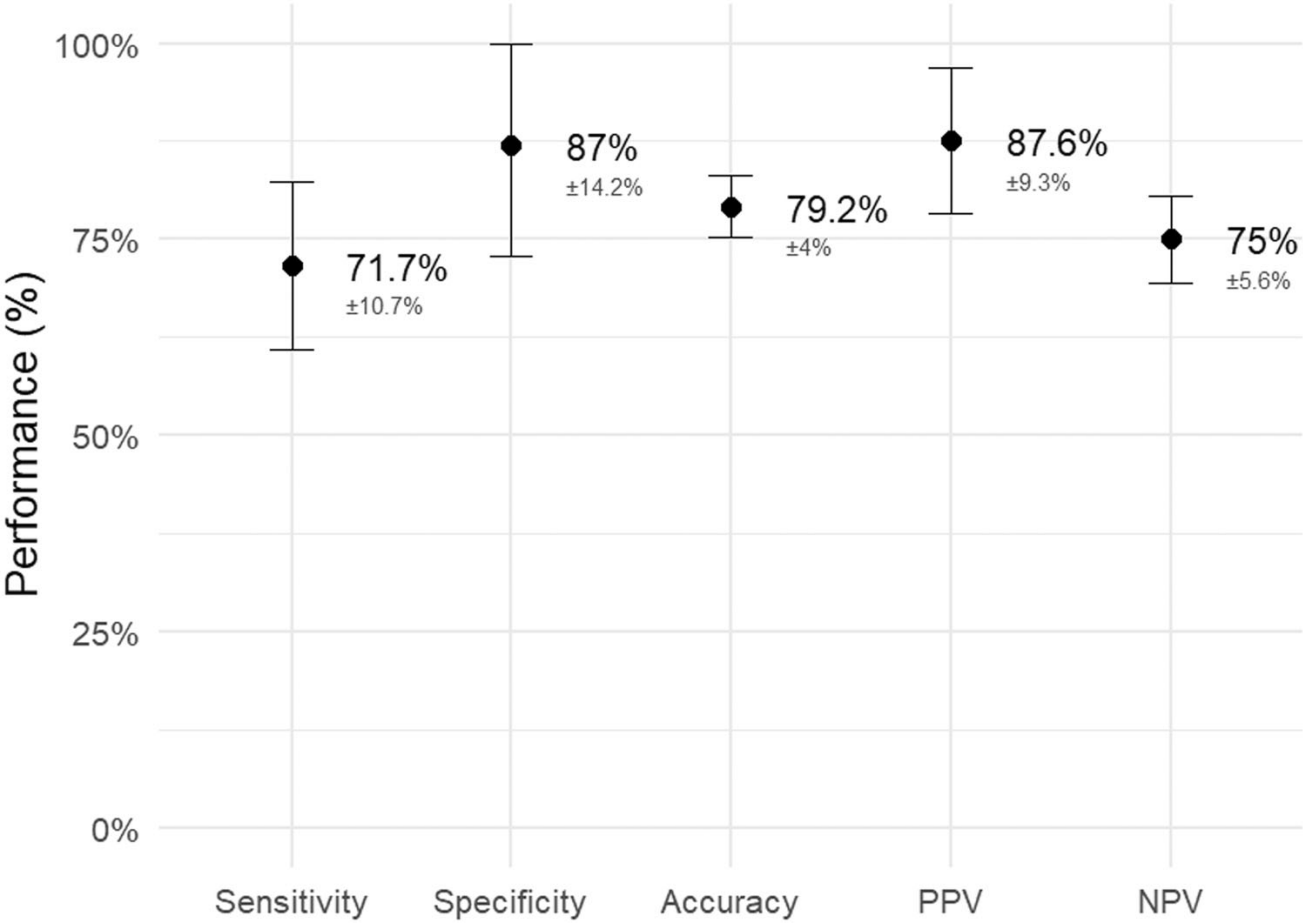
Performance of the algorithm measured as sensitivity, specificity, accuracy, positive predictive value (PPV) and negative predictive value (NPV) averaged over the 5 folds. Error bars present the standard deviations and mean, and standard deviation (SD) values are labelled next to the dots.

**Figure 3:**
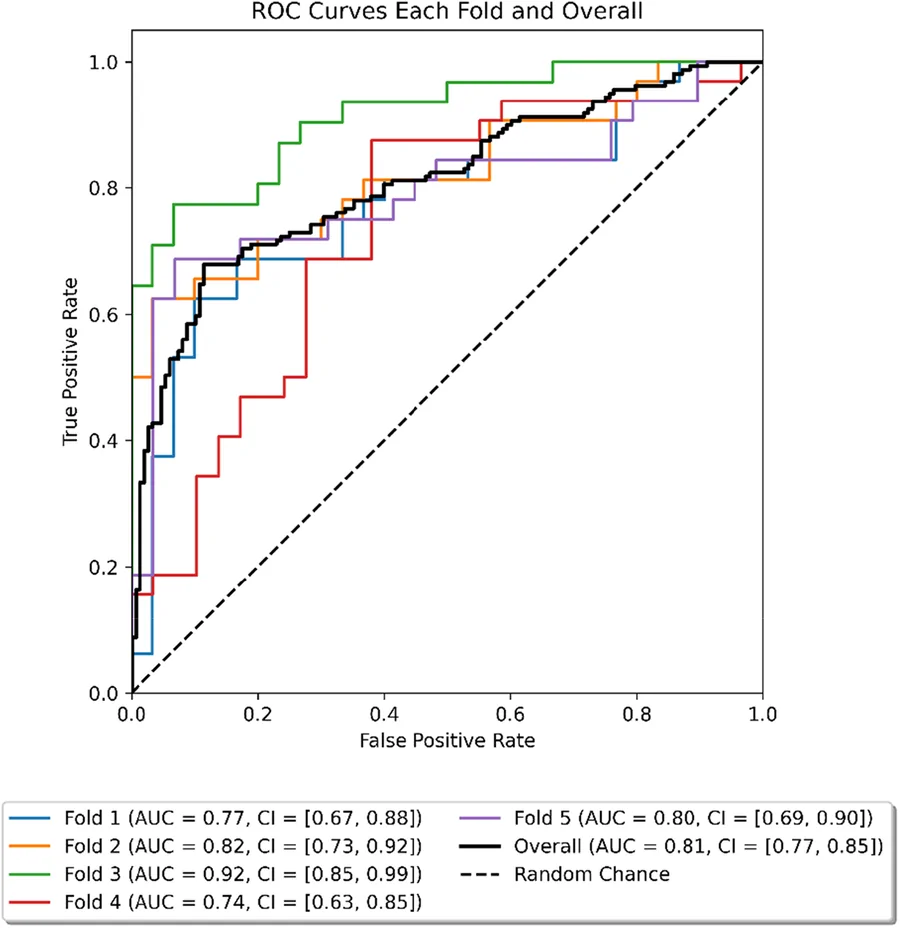
The receiver operating characteristic (ROC) curves of all fivefolds separately, as well as the overall ROC curve in black. AUC, area under the curve; CI, confidence interval.

The model achieved sensitivities between 62.5% and 87.5%, specificities between 62.1% and 96.7%, and accuracies between 75.4% and 85.2% across five test folds. The average performance measures (±SD) are presented in Figure 2. Confusion matrices with optimal cut‐off values for each fold are provided in Supporting Information S1: Tables S1–S5. The model demonstrated moderate‐to‐good discriminative ability, with an overall AUC of 0.81 (Figure 3).

### Various implant types

**Figure 4:**
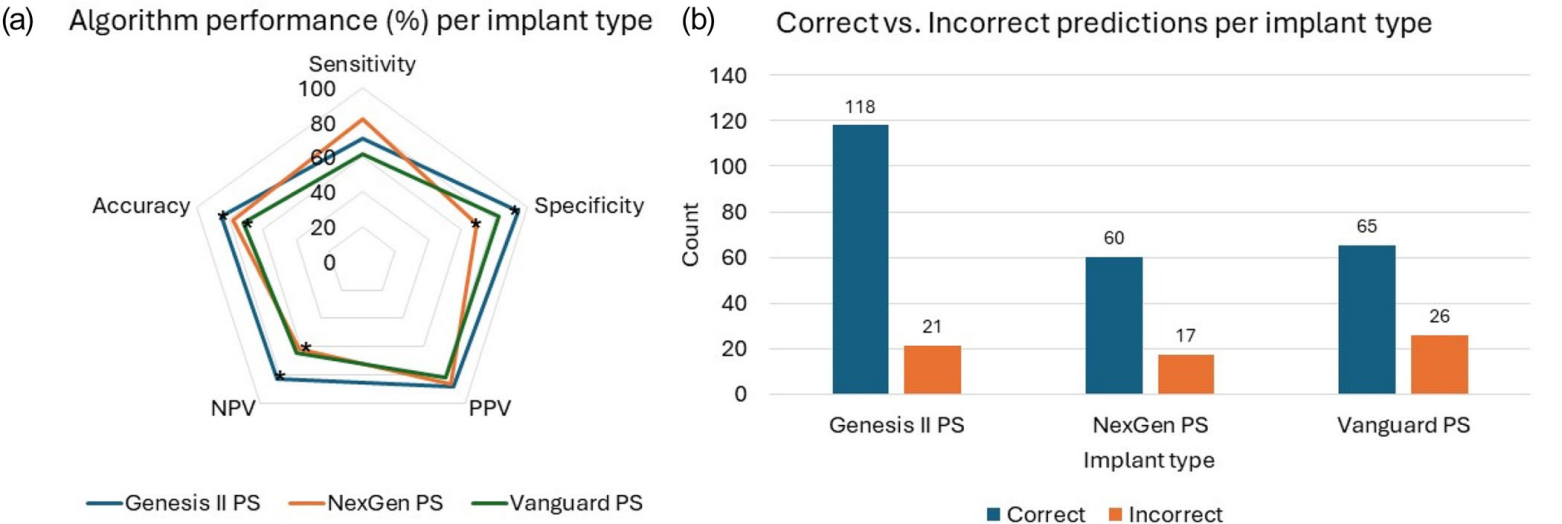
(a) The algorithm performance (sensitivity, specificity, accuracy, positive predictive value (PPV) and negative predictive value (NPV) for each implant type, with asterisk (*) indicating significant differences between these implant types. (b) The amount of correct and incorrect predictions per implant type. PS, posterior stabilized.

The distribution of implant types across folds did not differ significantly (*p* = 0.56). Similarly, the distribution of loose and fixed cases within implant groups was not significantly different (*p* > 0.39). In contrast, the overall distribution of loose versus fixed cases differed significantly across implant types (*p* < 0.01). Supporting Information S1: Table S6 provides a complete overview of the distribution of all implants across folds and groups. The number of correct predictions by the algorithm differed slightly across implant types (*p* = 0.05; Figure 4b).

After Bonferroni corrections, specificity was higher for Genesis II PS compared with NexGen PS (*p* < 0.01), whereas accuracy and NPV were higher for Genesis II PS compared with Vanguard PS (*p* = 0.04 and *p* = 0.03) (Figure 4a).

### Comparison with radiological reports

**Figure 5:**
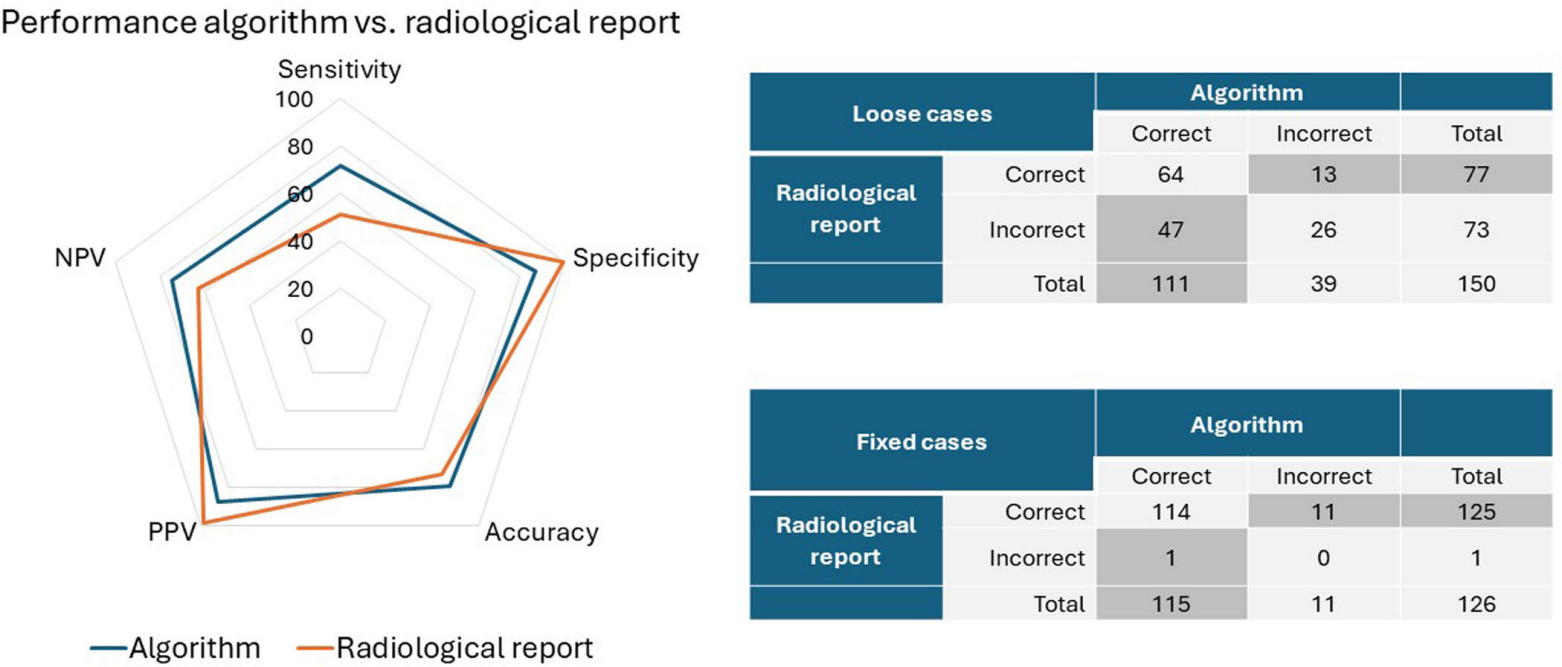
The radar plot on the left presents the algorithm's performance compared to the radiological report performance. The tables on the right present the algorithm's predictions compared to the radiological report predictions for all loose (upper table) and fixed (lower table) cases. The grey areas highlight the cases in which the algorithm's prediction differed from the radiological report prediction. NPV, negative predictive value.

Radiological reports achieved a sensitivity of 51.3%, specificity of 99.2% and accuracy of 73.2% (Supporting Information S1: Table S7). A total of 31 cases lacked report data (22 fixed and 9 loose). The algorithm correctly identified 47 loose cases in which the radiological report predictions resulted in false negatives. In contrast, the reports correctly identified 13 cases in which the algorithm was incorrect (Figure 5). For the fixed cases, the algorithm detected one case missed in the radiological reports, while the radiological report detected 11 cases missed by the algorithm (Figure 5). Overall, McNemar's test indicated that the algorithm outperformed the radiological reports (*χ* ^2^(1) = 8.00, *p* = 0.005).

### Visual explainability

**Figure 6:**
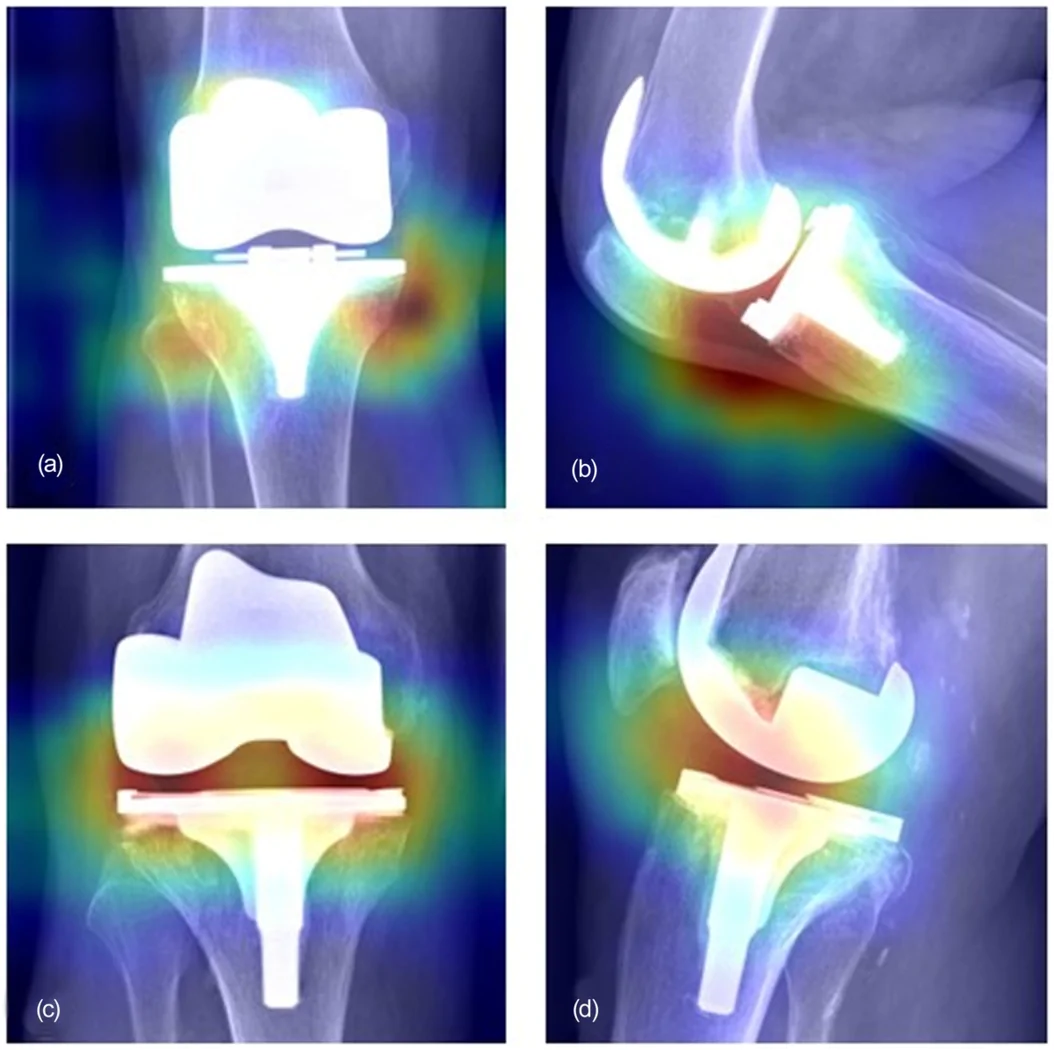
Grad‐CAM visualizations of a correctly classified loosening case (a) anteroposterior image and (b) lateral image and fixed case (c) anteroposterior image and (d) lateral image. Grad‐CAM, Gradient‐weighted Class Activation Mapping.

Grad‐CAM visualizations revealed that the model predominantly focused on anatomically relevant regions, particularly the bone–cement and implant–bone interfaces, in both AP and lateral projections. Representative Grad‐CAM visualizations are shown in Figure 6 for both a correctly classified loose case (a and b) and a fixed case (c and d). The full set of Grad‐CAM visualizations across all folds is provided in Supporting Information S1: Figures S1–S10. It should be noted that Grad‐CAM is a tool, and its images highlight the areas that were most influential for the model's decision. Therefore, a heatmap colouring can sometimes appear inverted, since the final layer of the network can produce both positive and negative activations for a given class.

## DISCUSSION

This proof‐of‐concept study demonstrates the potential of DL to transform the detection of implant loosening in TKA, addressing a critical challenge in orthopaedic diagnostics. The developed algorithm identifies radiographic features associated with loosening, indicating that it may detect subtle patterns often missed during routine assessment. Such automated detection could offer an important advantage, as current detection methods rely on subjective evaluation and are limited by the constraints of 2D imaging or the need for an additional CT scan, which increases radiation exposure and healthcare costs. Notably, the algorithm outperformed standard radiological evaluations, highlighting its potential to improve the diagnostic process.

Previous studies have explored similar DL approaches. Shah et al. [19] investigated DL models for detecting implant loosening in knee and hip arthroplasty, using intraoperative fixation status during revision surgery as the gold standard. Similar to the present method, they used both segmented AP and ML imaging in their DL model. Their model achieved an accuracy of over 80% for both TKAs and total hip arthroplasties (THAs), with the DenseNet model reaching up to 95.3% accuracy (92.2% for Inception) when incorporating historical patient data. Their results, without preoperative patient information but for TKA and THA combined, are slightly higher than our highest accuracy score of 85.2%. For TKAs specifically, Shah et al. reported a sensitivity of 69.8%, specificity of 95.2%, and accuracy of 85.8%, versus our average of 71.7%, 87.0% and 79.2%, respectively. Their model incorporated 32 preoperative patient variables, which likely contributed to its higher performance, whereas our model used imaging data alone. Details regarding implant types and dataset composition were limited in their report, and their TKA dataset was relatively small with an imbalance in fixation status between THAs and TKAs. Similarly, Lau et al. reported 96.3% accuracy in TKA loosening detection, but dataset and methodological details were sparse [15]. Unlike Shah et al., they found no additional benefit in incorporating clinical patient data into the model. Attempts to reproduce their results using our best approximation of their methodology were unsuccessful. Furthermore, neither study reported cut‐off values, which limits direct comparability.

In the present cohort, specificity was slightly lower for the NexGen PS implant. This may be attributed to its higher prevalence of loosening. At the clinic, NexGen PS implants are not routinely used, meaning that patients with these implants often sought second opinions for complications. Additionally, a specific PS type of this implant has been associated with a significantly higher revision rate for both septic and aseptic loosening, which has prompted continued implant surveillance [12]. This emphasizes the importance of considering implant‐specific characteristics when developing and implementing DL models. Future iterations should incorporate implant metadata to enhance accuracy and robustness. Post hoc analysis indicated that the algorithm performed best in Genesis PS cases, which may be explained by the fact that this group represented the largest implant cohort, providing more data for model learning and evaluation.

Comparison with routine radiological reports revealed complementary strengths. Routine assessments achieved high specificity but low sensitivity. The algorithm detected significantly more loosening cases, though it also produced some false positives. These findings underscore the subjectivity and variability inherent in routine radiological assessments, particularly when subtle indicators are present. Radiologists maintain high specificity, but their reliance on overt signs may lead to underdiagnosis of early‐stage loosening. The algorithm appears to improve sensitivity, particularly in cases that would otherwise be overlooked. These results highlight the complementary strengths of algorithmic analysis and expert radiological evaluation. A collaborative approach could optimize diagnostic accuracy by ensuring both early detection and appropriate human oversight to minimize false positives. It should be emphasized, however, that this comparison with the radiological report is exploratory and does not constitute a definitive benchmark of radiologist performance.

This study employed fivefold cross‐validation, with fold‐specific cut‐off values chosen to maximize Youden's index. For clinical use, cut‐offs should be tailored to suit the intended task [9]. For example, when the DL tool is primarily intended to exclude loosening, prioritizing specificity would be advisable. Conversely, if the tool is intended for definitive diagnosis, prioritizing PPV would be advisable. Therefore, as the tool advances toward clinical implementation, close consultation with clinicians will be essential to define optimal thresholds.

This study has some limitations that should be considered when interpreting the results. First, the study was conducted retrospectively, which may introduce missing data and affect radiologist performance. However, this approach closely reflects real‐world clinical practice, as radiologists naturally assess implant fixation without being explicitly instructed to focus on loosening. Requesting a reassessment with loosening in mind could lead to overemphasis, which would not accurately represent a typical diagnostic scenario. Second, given the exploratory nature of this study, only explicit loosening or clear fixation cases were included, which limits the variation. Future research should incorporate the ‘grey areas’ to enhance the model's applicability. Third, the predominantly single‐centre dataset, with some external cases, limits the generalizability of the findings. Differences in imaging protocols, patient demographics, and implant distributions across centres could affect algorithm performance. Addressing these limitations through prospective, multicentre studies will be essential. Lastly, since both the AP and lateral radiographs were processed through a shared convolutional backbone, the resulting Grad‐CAMs reflect a combined feature representation. This architectural choice limits direct interpretation of activations.

The ultimate goal is to integrate this tool into clinical practice, using it as a tool to assist orthopaedic surgeons and radiologists in making informed decisions. To achieve this goal, the algorithm requires further development. Given the proof‐of‐concept nature of the study, the algorithm was built around the well‐established InceptionV3 architecture, which is a strong‐performing algorithm for classification tasks. During the exploratory phase, networks such as DenseNet, Xception and InceptionResNetV2 were also tested for this specific challenge. InceptionV3 proved to be the most suitable model for this task. A pre‐trained InceptionV3 model was also explored, but did not yield better results on the dataset. Currently, no pre‐trained models specifically tailored for implant loosening detection are available. Existing foundation models that included radiographs of any type were optimized for lower image resolution (256 × 256 pixels), which is not suitable for the 512 × 512 images used in this study, which were considered the lower limit for these radiological images. However, there may be greater potential using different or custom networks and the inclusion of historical patient data. Recent research in musculoskeletal imaging supports this approach: Hassan et al. [10] demonstrated that a custom‐designed convolutional neural network outperformed several popular transfer learning models in fracture detection from radiographs. This emphasizes that lightweight, task‐specific model architectures can be highly effective even with limited or imbalanced datasets.

In addition, object‐detection frameworks could offer a promising direction for future work. Recent research by Ariyametkul et al. [2] demonstrated that detection‐based architectures, such as You Only Look Once (YOLO), can provide more spatially explicit and interpretable explanations in medical imaging tasks. Unlike classification networks, these models inherently learn spatial localization. This approach could enhance both interpretability and performance when applied to multi‐view radiographic data.

Broader reviews of AI in healthcare underline the importance of improving model interpretability for clinical adoption. Ennab et al. [6] emphasized that the trade‐off between accuracy and transparency remains a key challenge, and that future AI systems should integrate explainability, uncertainty estimation and user‐centred design to ensure trust and safety in clinical decision‐making. These insights align with the present study's aim to advance beyond classification towards architectures that combine accurate prediction with interpretable spatial localization. One of the key next steps will therefore be the development of a customized algorithm, tailored to the unique characteristics of this dataset and task. This algorithm should subsequently be trained and tested on larger, multicentre datasets to achieve external validation [14, 18].

By providing consistent and accurate predictions, the DL tool has the potential to transform the standard of care in knee arthroplasty, enabling earlier detection of implant loosening, reducing diagnostic delays, and ultimately improving patient outcomes. After further improvements, the tool could be integrated into clinical workflows in various ways. One potential use case is as a triage system that automatically flags radiographs with suspected loosening for closer review. Alternatively, it could serve as a second reader, aiding the radiologist in the assessment. However, the most suitable integration strategy will depend on the clinical context. For instance, high specificity would be essential if the tool is used to exclude loosening in patients with unclear symptoms, whereas higher sensitivity may be preferable when early detection is the priority. False positives would, in practice, be filtered through radiologist and orthopaedic review. False negatives remain a limitation, underscoring the importance of using the tool as an aid rather than a replacement for expert evaluation.

In conclusion, this study supports the feasibility of DL to detect implant loosening in TKA. This algorithm can identify predictive radiographic patterns that may be missed during routine radiographic assessment. With further development and validation, this tool has the potential to enhance diagnostic precision, streamline clinical workflows, and improve outcomes in knee arthroplasty.

## AUTHOR CONTRIBUTIONS

Kelly Mills was responsible for the methodology, validation, formal analysis, data curation, original draft writing, visualization and project administration. Guido A. de Jong contributed to the methodology, software development, validation, formal analysis, writing—review and editing, and visualization. Simon N. van Laarhoven was involved in the conceptualization, provided resources, contributed to data curation, and writing—review and editing. Thomas J. J. Maal contributed to the conceptualization, software, writing—review and editing, and supervision. Petra J. C. Heesterbeek was responsible for the conceptualization, methodology, validation, writing—review and editing, and supervision.

## CONFLICT OF INTEREST STATEMENT

Petra J. C. Heesterbeek reports an unpaid board membership of the International RSA Society and the International Society for Technology in Arthroplasty (ISTA). Simon N. van Laarhoven reports receiving consulting fees from Smith & Nephew. The remaining authors declare no conflict of interest.

## ETHICS STATEMENT

Informed consent was not required for this retrospective case study. All data were anonymized and handled in accordance with institutional and ethical guidelines.

## Supporting information

Supplementary Materials_Clean.

## Data Availability

The data that support the findings of this study are available on request from the corresponding author. The data are not publicly available due to privacy or ethical restrictions.

## References

[1] Achakri H, Ben‐Shlomo Y, Blom A, Boulton C, Bridgens J, Brittain R, et al. The National Joint Registry 20th Annual Report 2023. London: National Joint Registry; 2023. 38422195

[2] Ariyametkul A , Paing MP . Analyzing explainability of YOLO‐based breast cancer detection using heat map visualizations. Quant Imaging Med Surg. 2025;15:6252–6271. 10.21037/qims-2024-2911 PMC12290753 40727375

[3] Arndt KB , Schrøder HM , Troelsen A , Lindberg‐Larsen M . Patient‐reported outcomes and satisfaction after revisions of medial unicompartmental knee arthroplasties for unexplained pain vs aseptic loosening. Knee Surg Sports Traumatol Arthrosc. 2023;31:4766–4772. 10.1007/s00167-023-07483-z PMC10598095 37498328

[4] Batailler C , Shatrov J , Sappey‐Marinier E , Servien E , Parratte S , Lustig S . Artificial intelligence in knee arthroplasty: current concept of the available clinical applications. Arthroplasty. 2022;4:17. 10.1186/s42836-022-00119-6 PMC9059406 35491420

[5] Dalury DF , Pomeroy DL , Gorab RS , Adams MJ . Why are total knee arthroplasties being revised? J Arthroplasty. 2013;28:120–121. 10.1016/j.arth.2013.04.051 23886410

[6] Ennab M , Mcheick H . Enhancing interpretability and accuracy of AI models in healthcare: a comprehensive review on challenges and future directions. Front Robot AI. 2024;11:1444763. 10.3389/frobt.2024.1444763 PMC11638409 39677978

[7] Goharian A, Golkar E. Chapter one—general concepts of interactions of bone with orthopedic implants and possible failures. In: Interactions of Bone with Orthopedic Implants and Possible Failures. Oxford (UK): Academic Press; 2022. p. 1–31.

[8] Greidanus NV , Peterson RC , Masri BA , Garbuz DS . Quality of life outcomes in revision versus primary total knee arthroplasty. J Arthroplasty. 2011;26:615–620. 10.1016/j.arth.2010.04.026 20541360

[9] Habibzadeh F , Habibzadeh P , Yadollahie M . On determining the most appropriate test cut‐off value: the case of tests with continuous results. Biochem Med. 2016;26:297–307. 10.11613/BM.2016.034 PMC5082211 27812299

[10] Hassan A , Afzaal I , Muneeb N , Batool A , Noor H . Fracture detection in x‐rays using custom CNN and transfer learning models. J Med Imaging AI. 2025;12:123–130.

[11] Hirschmann MT , Konala P , Iranpour F , Kerner A , Rasch H , Friederich NF . Clinical value of SPECT/CT for evaluation of patients with painful knees after total knee arthroplasty—a new dimension of diagnostics? BMC Musculoskelet Disord. 2011;12:36. 10.1186/1471-2474-12-36 PMC3040164 21294878

[12] Keohane D , Sheridan GA , Masterson E . High rate of tibial debonding and failure in a popular knee replacement: a follow‐up review. Bone Joint Open. 2022;3:495–501. 10.1302/2633-1462.36.BJO-2022-0043.R1 PMC9233423 35698801

[13] Klug A , Gramlich Y , Rudert M , Drees P , Hoffmann R , Weißenberger M , et al. The projected volume of primary and revision total knee arthroplasty will place an immense burden on future health care systems over the next 30 years. Knee Surg Sports Traumatol Arthrosc. 2021;29:3287–3298. 10.1007/s00167-020-06154-7 PMC7362328 32671435

[14] Krois J , Garcia Cantu A , Chaurasia A , Patil R , Chaudhari PK , Gaudin R , et al. Generalizability of deep learning models for dental image analysis. Sci Rep. 2021;11:6102. 10.1038/s41598-021-85454-5 PMC7969919 33731732

[15] Lau LCM , Chui ECS , Man GCW , Xin Y , Ho KKW , Mak KKK , et al. A novel image‐based machine learning model with superior accuracy and predictability for knee arthroplasty loosening detection and clinical decision making. J Orthop Translat. 2022;36:177–183. 10.1016/j.jot.2022.07.004 PMC9562957 36263380

[16] McNemar Q . Note on the sampling error of the difference between correlated proportions or percentages. Psychometrika. 1947;12:153–157. 10.1007/BF02295996 20254758

[17] Postler A , Lützner C , Beyer F , Tille E , Lützner J . Analysis of total knee arthroplasty revision causes. BMC Musculoskelet Disord. 2018;19:55. 10.1186/s12891-018-1977-y PMC5813428 29444666

[18] Rockenschaub P , Hilbert A , Kossen T , Elbers P , Von Dincklage F , Madai VI , et al. The impact of multi‐institution datasets on the generalizability of machine learning prediction models in the ICU. Crit Care Med. 2024;52:1710–1721. 10.1097/CCM.0000000000006359 PMC11469625 38958568

[19] Shah RF , Bini SA , Martinez AM , Pedoia V , Vail TP . Incremental inputs improve the automated detection of implant loosening using machine‐learning algorithms. Bone Joint J. 2020;102–B:101–106. 10.1302/0301-620X.102B6.BJJ-2019-1577.R1 32475275

[20] Tejani AS , Klontzas ME , Gatti AA , Mongan JT , Moy L , Park SH , et al. Checklist for Artificial Intelligence in Medical Imaging (CLAIM): 2024 Update. Radiol Artif Intell. 2024;6:e240300. 10.1148/ryai.240300 PMC11304031 38809149

