# Supplementary material for: Deep learning applied to standard radiographs improves detection of implant loosening in total knee arthroplasty: A proof‐of‐concept study
Source: J Exp Orthop. 2026 Jan 19;13(1):e70611. doi: 10.1002/jeo2.70611 (PMC12813552; doi:10.1002/jeo2.70611)
Supplement: Supplementary file 1 — Supplementary Materials_Clean. [file JEO2-13-e70611-s001.pdf]

Supplementary Materials

Table 1: Confusion matrix presenting the algorithm's performance for fold 1. Sens: sensitivity; Spec: specificity; Acc: accuracy; PPV: positive predictive value; NPV: negative predictive value.

Fold 1 (cut-off: 0.52)		Predicted value		
		Loose	Fixed	
**Actual value**	Loose	20	12	Sens 62.5%
	Fixed	3	27	Spec 90.0%
		PPV 87.0%	NPV 69.2%	Acc 75.8%

Table 2: Confusion matrix presenting the algorithm's performance for fold 2. Sens: sensitivity; Spec: specificity; Acc: accuracy; PPV: positive predictive value; NPV: negative predictive value.

Fold 2 (cut-off: 0.51)		Predicted value		
		Loose	Fixed	
**Actual value**	Loose	20	12	Sens 62.5%
	Fixed	1	29	Spec 96.7%
		PPV 95.2%	NPV 70.7%	Acc 79.0%

Table 3: Confusion matrix presenting the algorithm's performance for fold 3. Sens: sensitivity; Spec: specificity; Acc: accuracy; PPV: positive predictive value; NPV: negative predictive value.

Fold 3 (cut-off: 0.11)		Predicted value		
		Loose	Fixed	
**Actual value**	Loose	24	7	Sens 77.4%
	Fixed	2	28	Spec 93.3%
		PPV 92.3%	NPV 80.0%	Acc 85.2%

Table 4: Confusion matrix presenting the algorithm's performance for fold 4. Sens: sensitivity; Spec: specificity; Acc: accuracy; PPV: positive predictive value; NPV: negative predictive value.

Fold 4 (cut-off: 0.51)		Predicted value		
		Loose	Fixed	
**Actual value**	Loose	28	4	Sens 87.5%
	Fixed	11	18	Spec 62.1%
		PPV 71.8%	NPV 81.8%	Acc 75.4%

Table 5: Confusion matrix presenting the algorithm's performance for fold 5. Sens: sensitivity; Spec: specificity; Acc: accuracy; PPV: positive predictive value; NPV: negative predictive value.

Fold 5 (cut-off: 0.32)		Predicted value		
		Loose	Fixed	
**Actual value**	Loose	22	10	Sens 68.8%
	Fixed	2	27	Spec 93.1%
		PPV 91.7%	NPV 73.0%	Acc 80.3%

Table 6: The number of cases per implant type per fold and in the overall dataset, presented as numbers of fixed/loose cases.

	Fold 1	Fold 2	Fold 3	Fold 4	Fold 5	Overall
**Genesis II PS[1]**	19/12	18/9	18/10	11/13	18/11	84/55
**NexGen PS[2]**	1/12	6/13	6/13	6/9	4/7	23/54
**Vanguard PS[2]**	10/8	6/10	6/8	12/10	7/14	41/50
**Total**	30/32	30/32	30/31	29/32	29/32	148/159

[1] Smith and Nephew, Memphis, USA; [2]Zimmer Biomet, Warsaw, USA

Table 7: Confusion matrix for radiological report prediction performance. Sens: sensitivity; Spec: specificity; Acc: accuracy; PPV: positive predictive value; NPV: negative predictive value.

Scenario 2		**Predicted value – radiological report**		
		Loose	Fixed	
**Actual value**	Loose	77	73	Sens 51.3%
	Fixed	1	125	Spec 99.2%
		PPV 98.7%	NPV 63.1%	Acc 73.2%

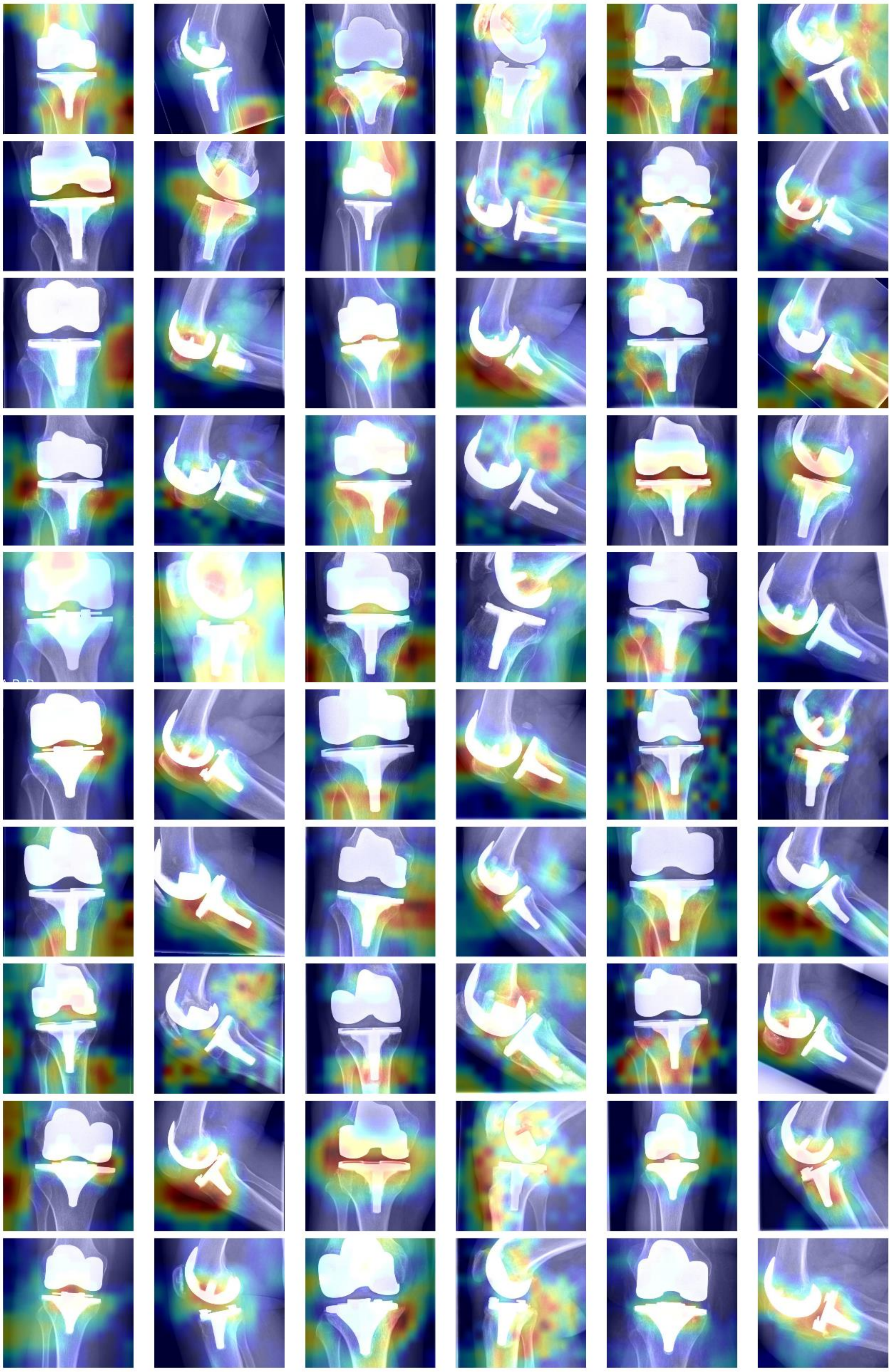

Figure 1: Gradient-weighted Class Activation Mapping (Grad-CAM) for all control cases in fold 1. Each panel shows the original anteroposterior and lateral radiographs alongside the corresponding Grad-CAM overlay.

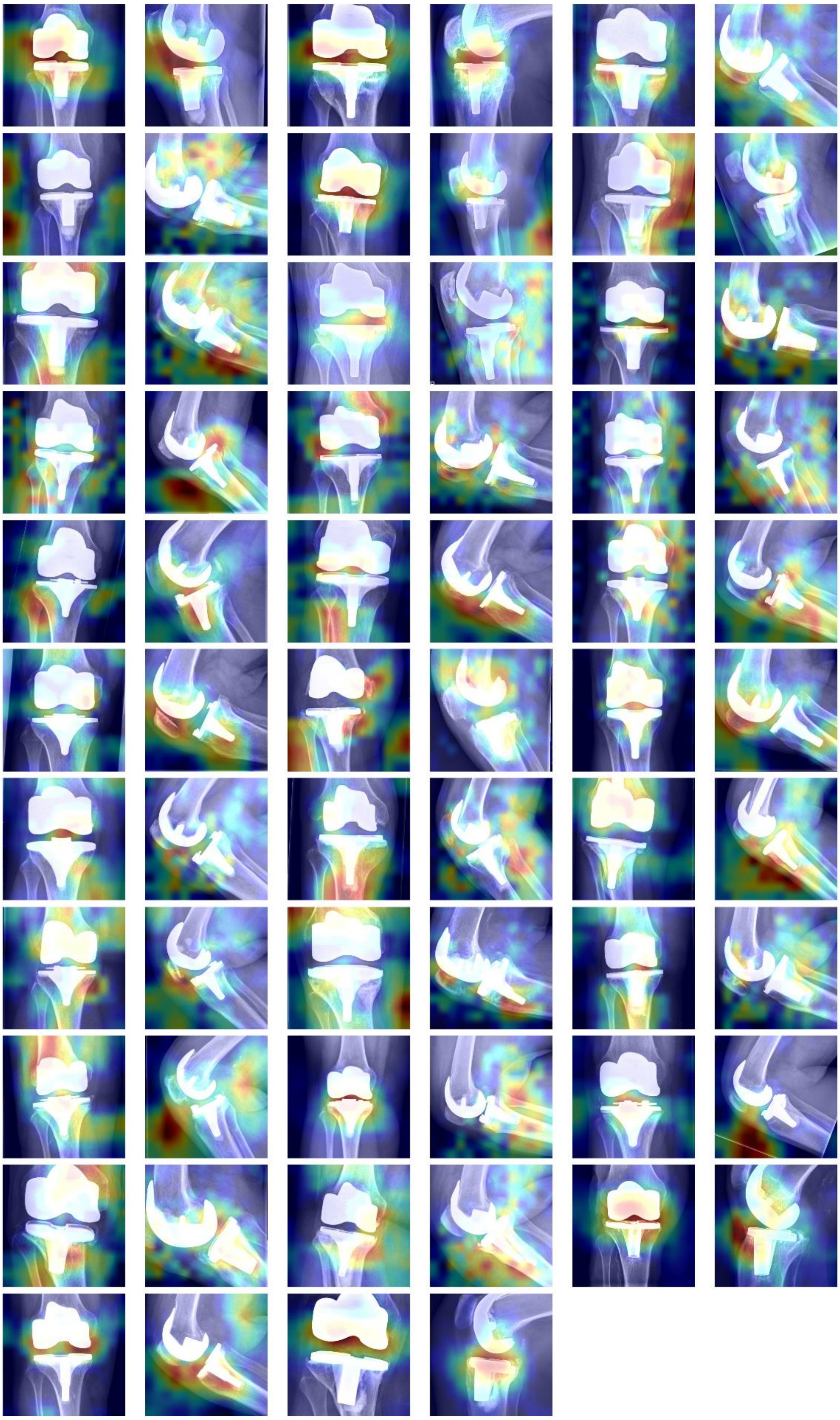

Figure 2: Gradient-weighted Class Activation Mapping (Grad-CAM) for all loosening cases in fold 1. Each panel shows the original anteroposterior and lateral radiographs alongside the corresponding Grad-CAM overlay.

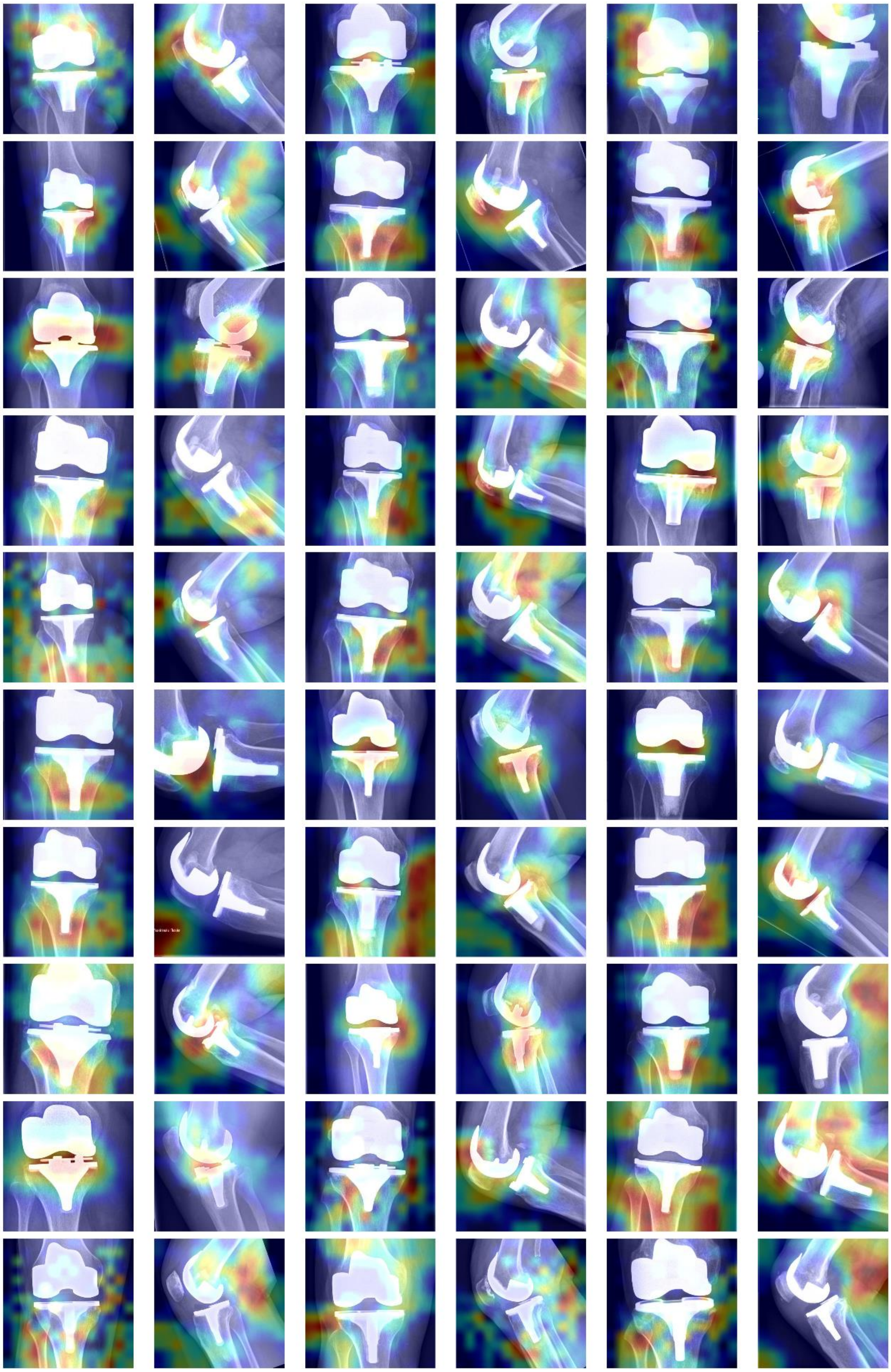

Figure 3: Gradient-weighted Class Activation Mapping (Grad-CAM) for all control cases in fold 2. Each panel shows the original anteroposterior and lateral radiographs alongside the corresponding Grad-CAM overlay.

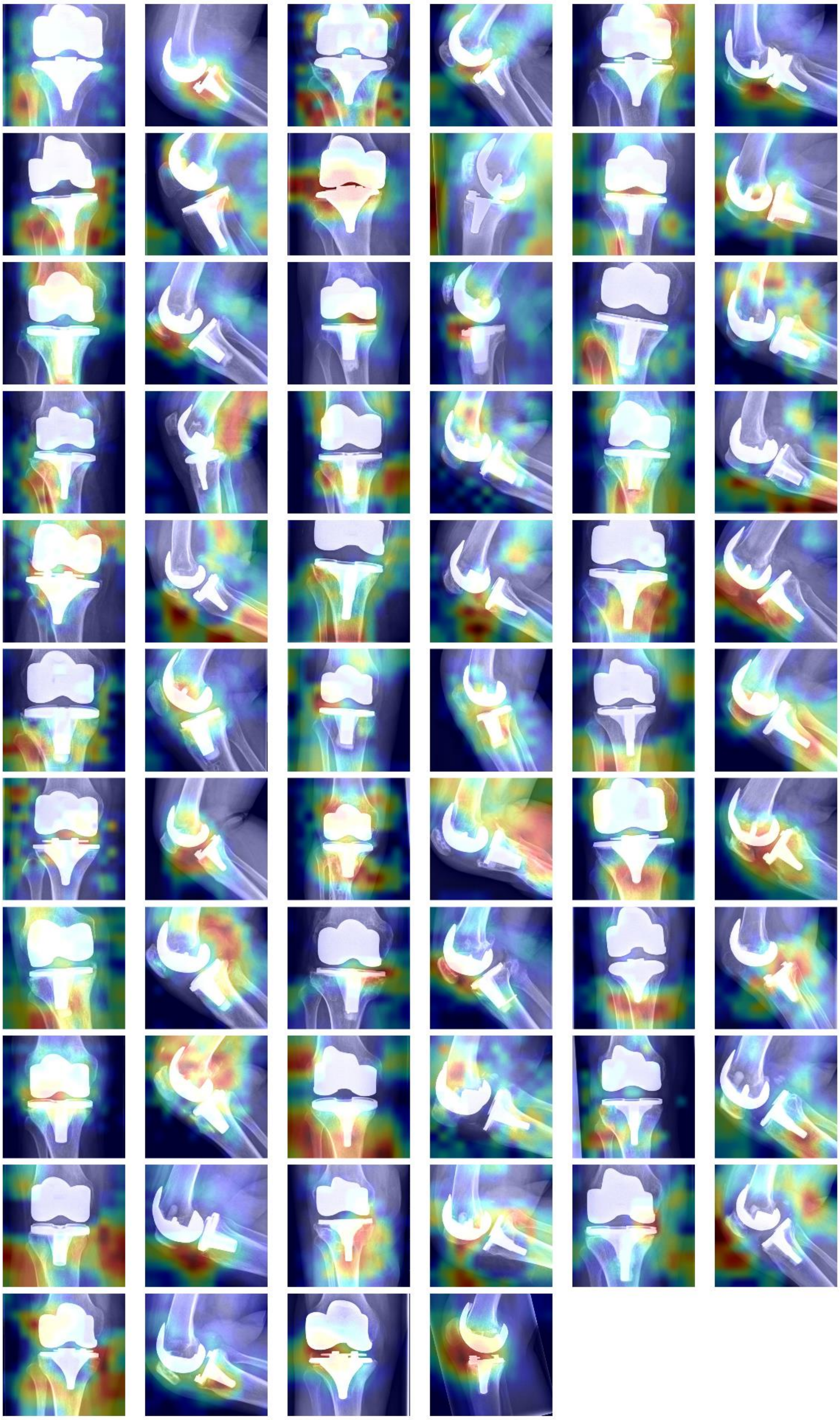

Figure 4: Gradient-weighted Class Activation Mapping (Grad-CAM) for all loosening cases in fold 2. Each panel shows the original anteroposterior and lateral radiographs alongside the corresponding Grad-CAM overlay.

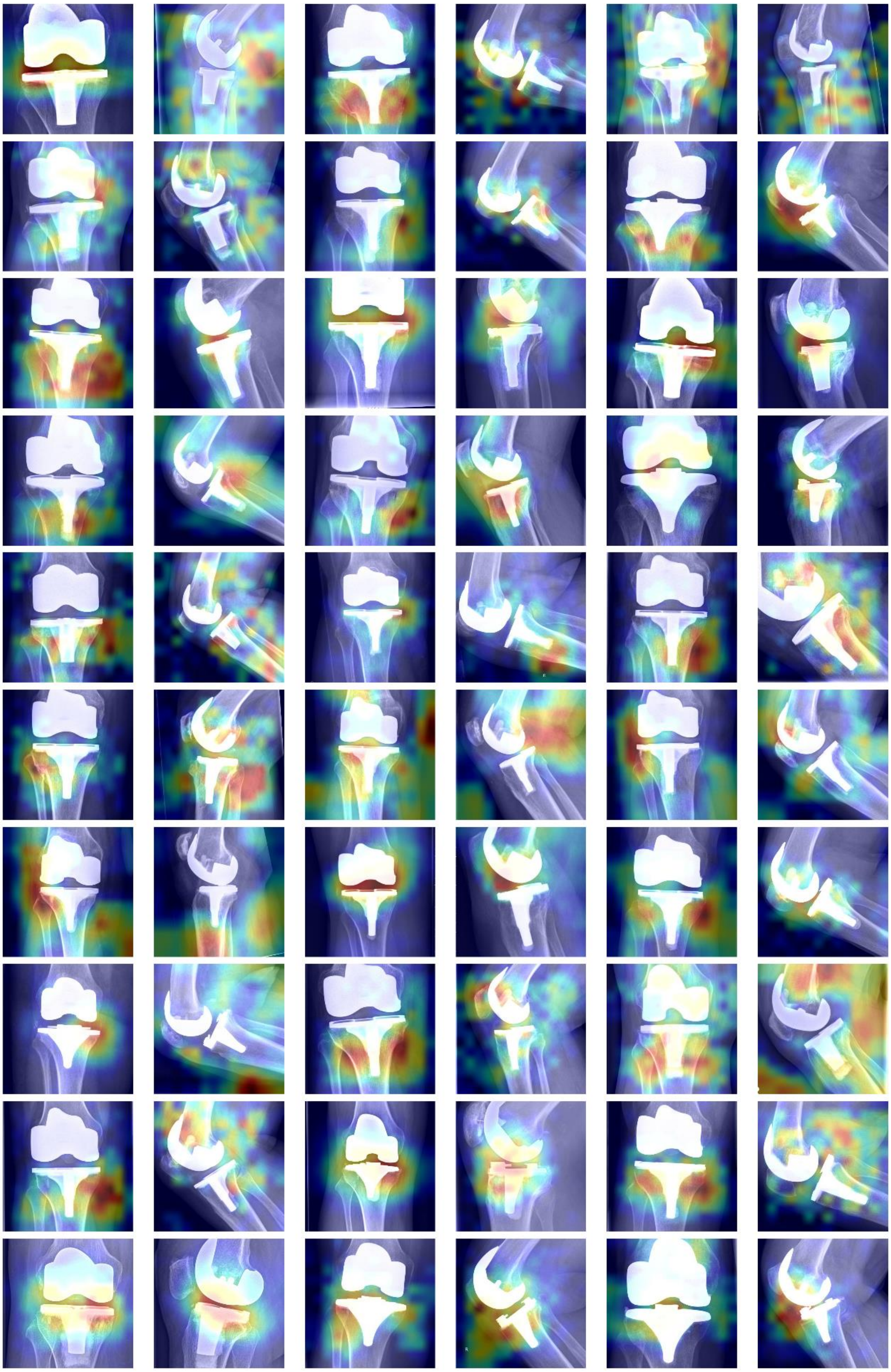

Figure 5: Gradient-weighted Class Activation Mapping (Grad-CAM) for all control cases in fold 3. Each panel shows the original anteroposterior and lateral radiographs alongside the corresponding Grad-CAM overlay.

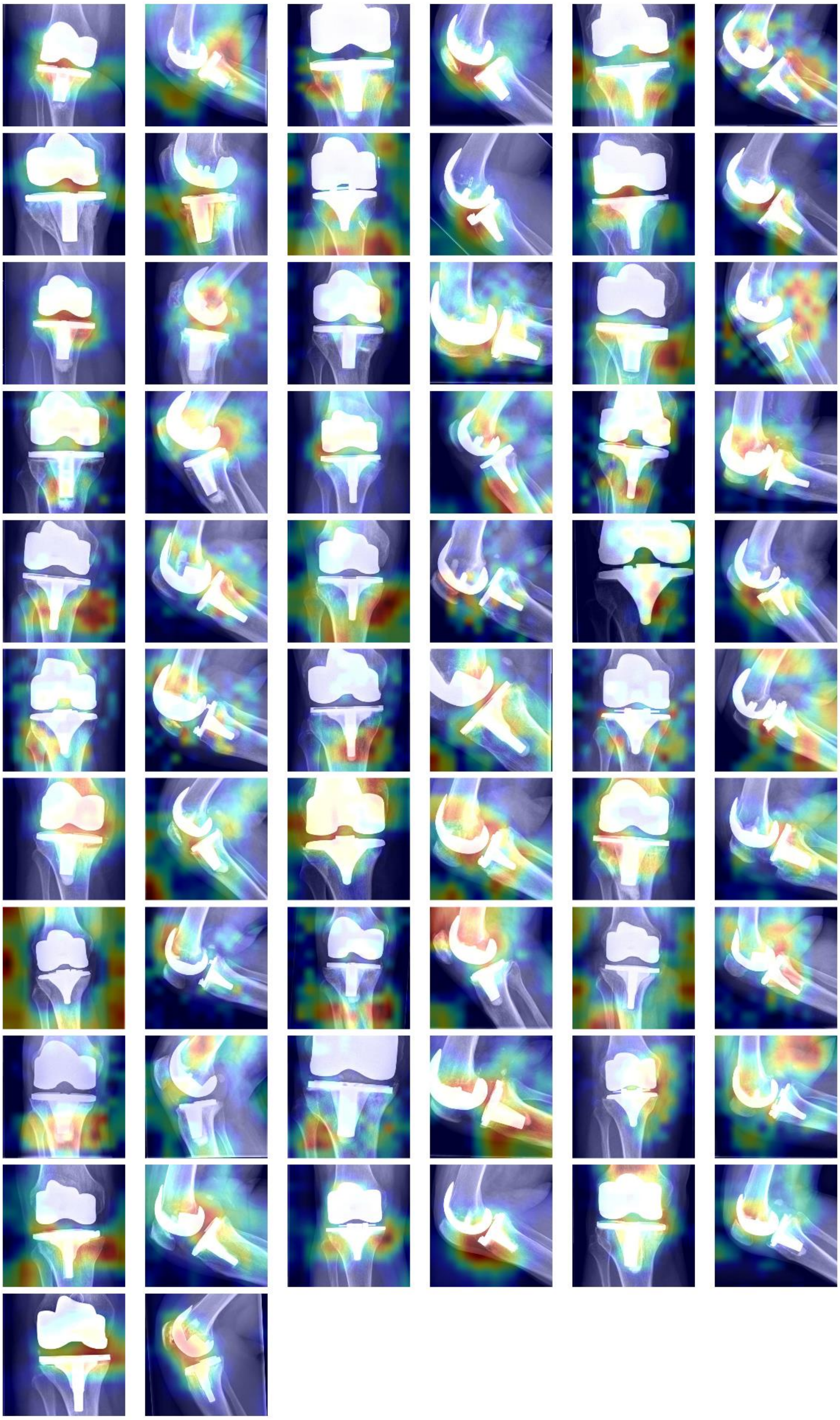

Figure 6: Gradient-weighted Class Activation Mapping (Grad-CAM) for all loosening cases in fold 3. Each panel shows the original anteroposterior and lateral radiographs alongside the corresponding Grad-CAM overlay.

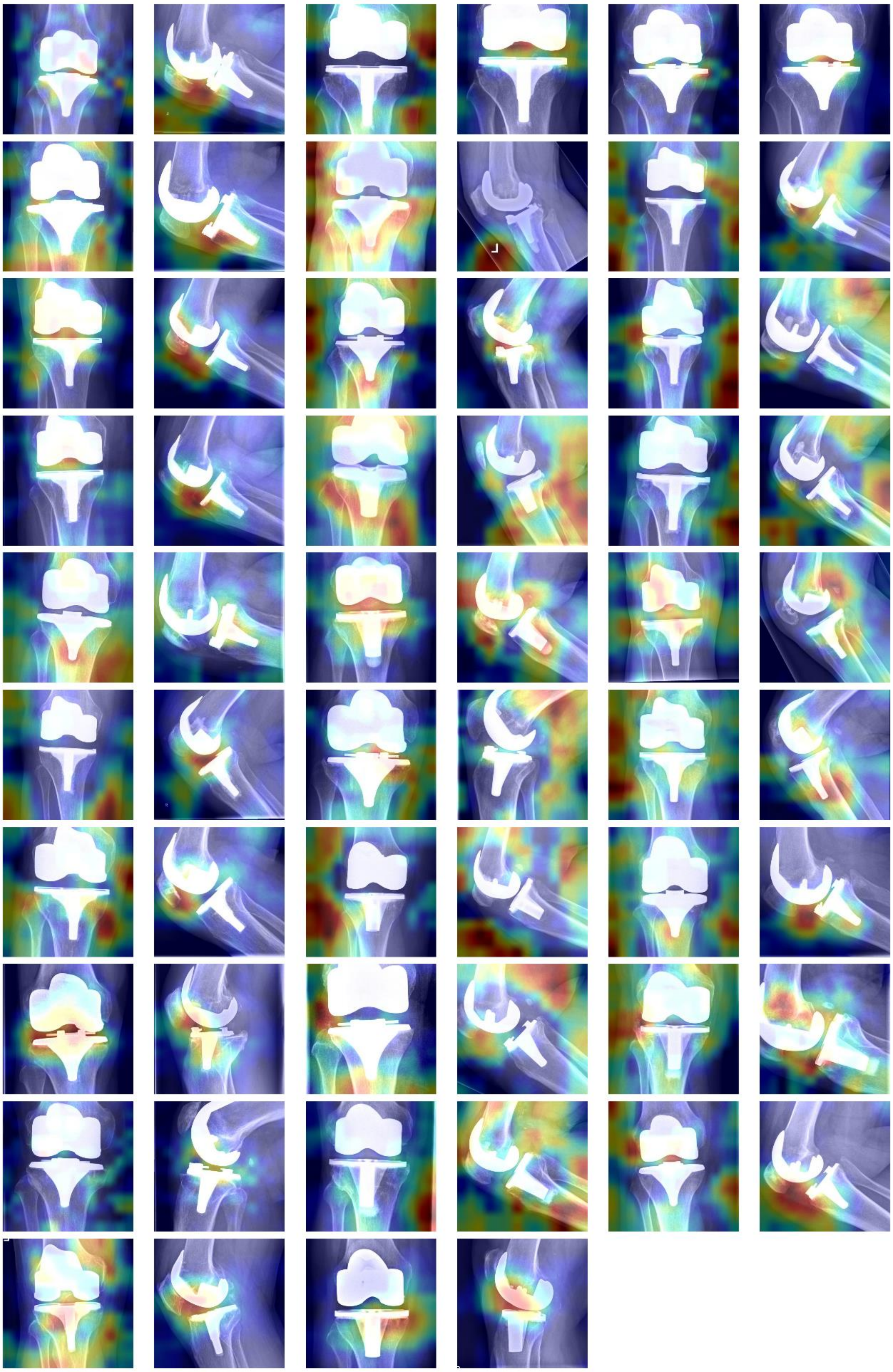

Figure 7: Gradient-weighted Class Activation Mapping (Grad-CAM) for all control cases in fold 4. Each panel shows the original anteroposterior and lateral radiographs alongside the corresponding Grad-CAM overlay.

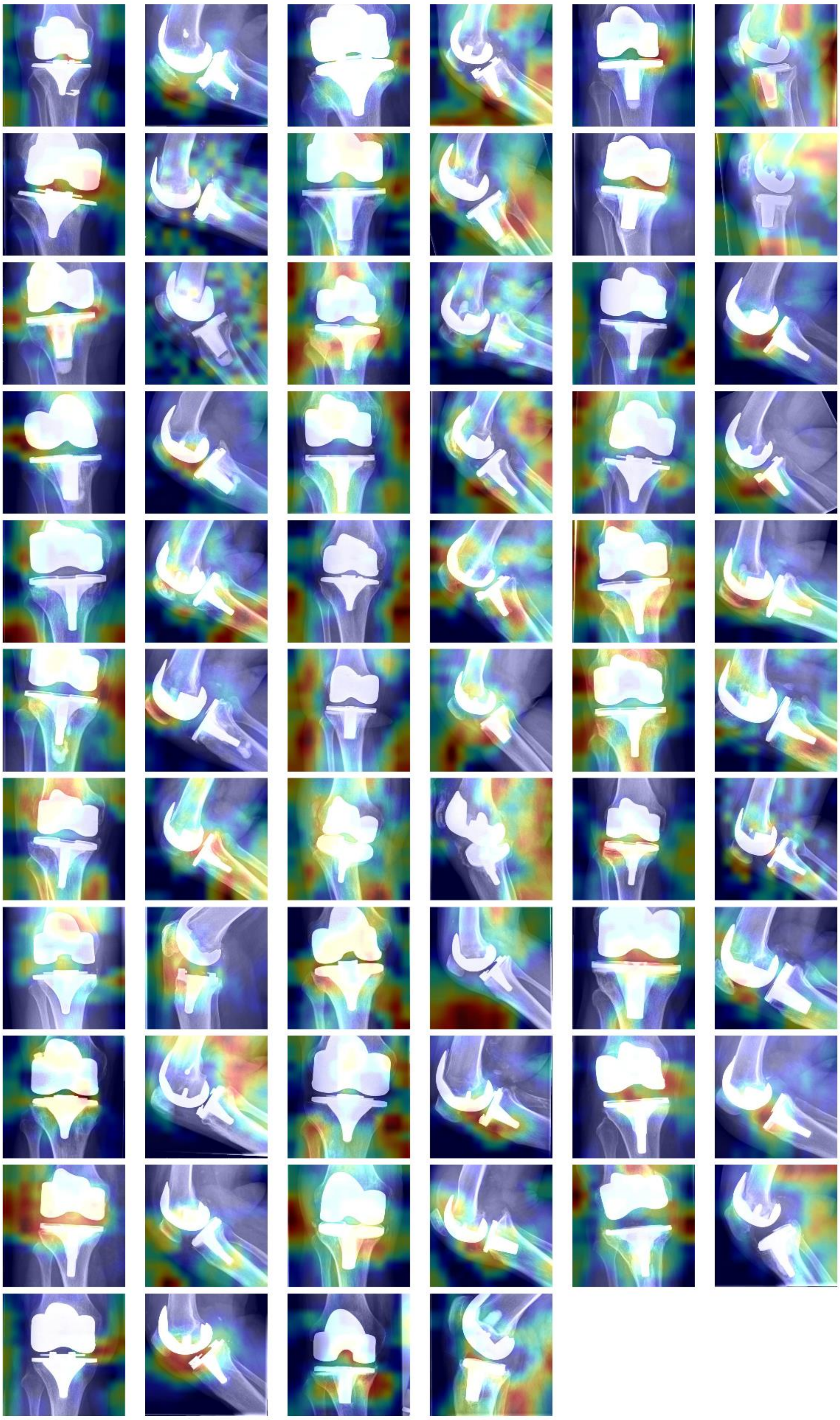

Figure 8: Gradient-weighted Class Activation Mapping (Grad-CAM) for all loosening cases in fold 4. Each panel shows the original anteroposterior and lateral radiographs alongside the corresponding Grad-CAM overlay.

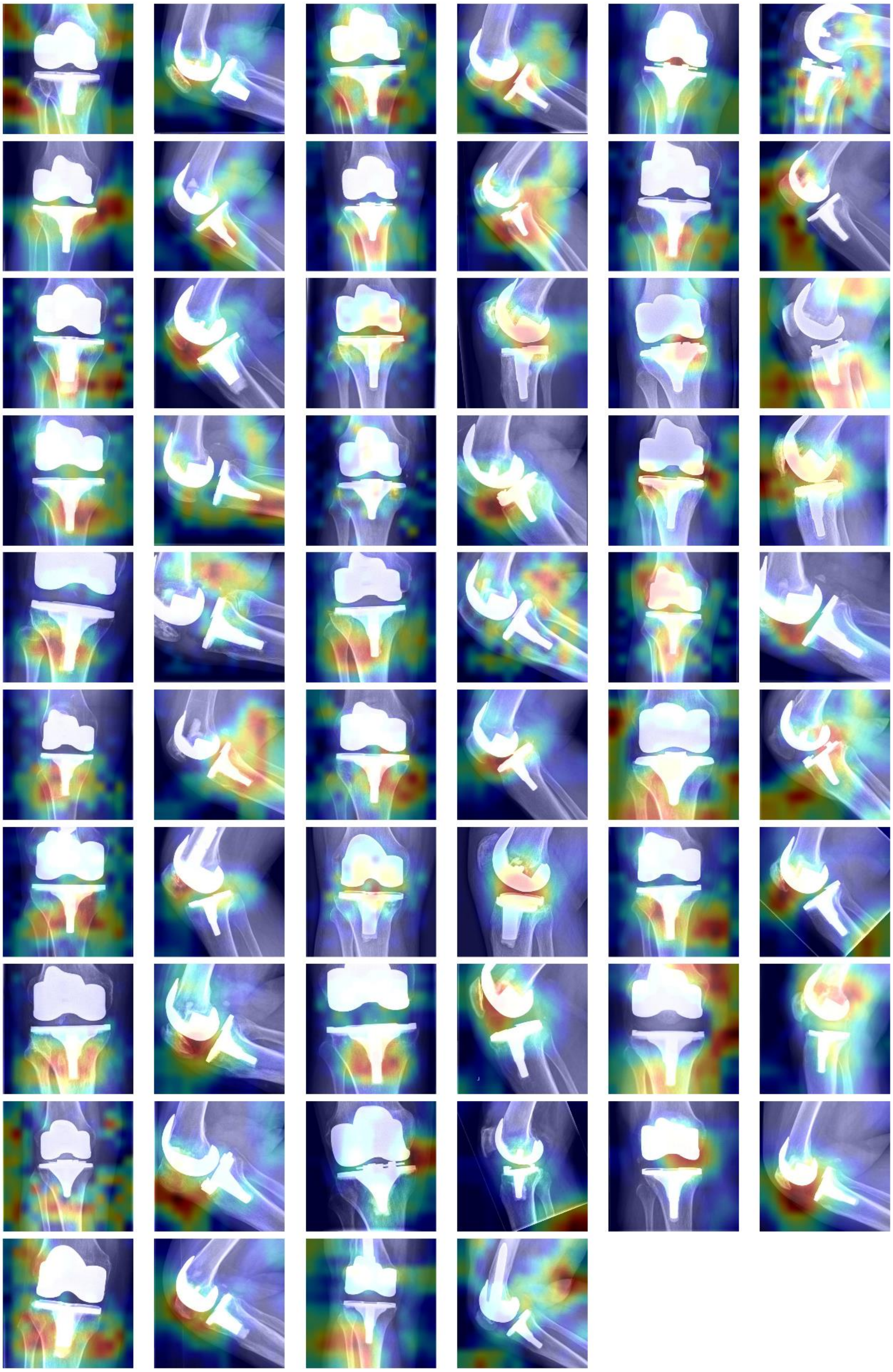

Figure 9: Gradient-weighted Class Activation Mapping (Grad-CAM) for all control cases in fold 5. Each panel shows the original anteroposterior and lateral radiographs alongside the corresponding Grad-CAM overlay.

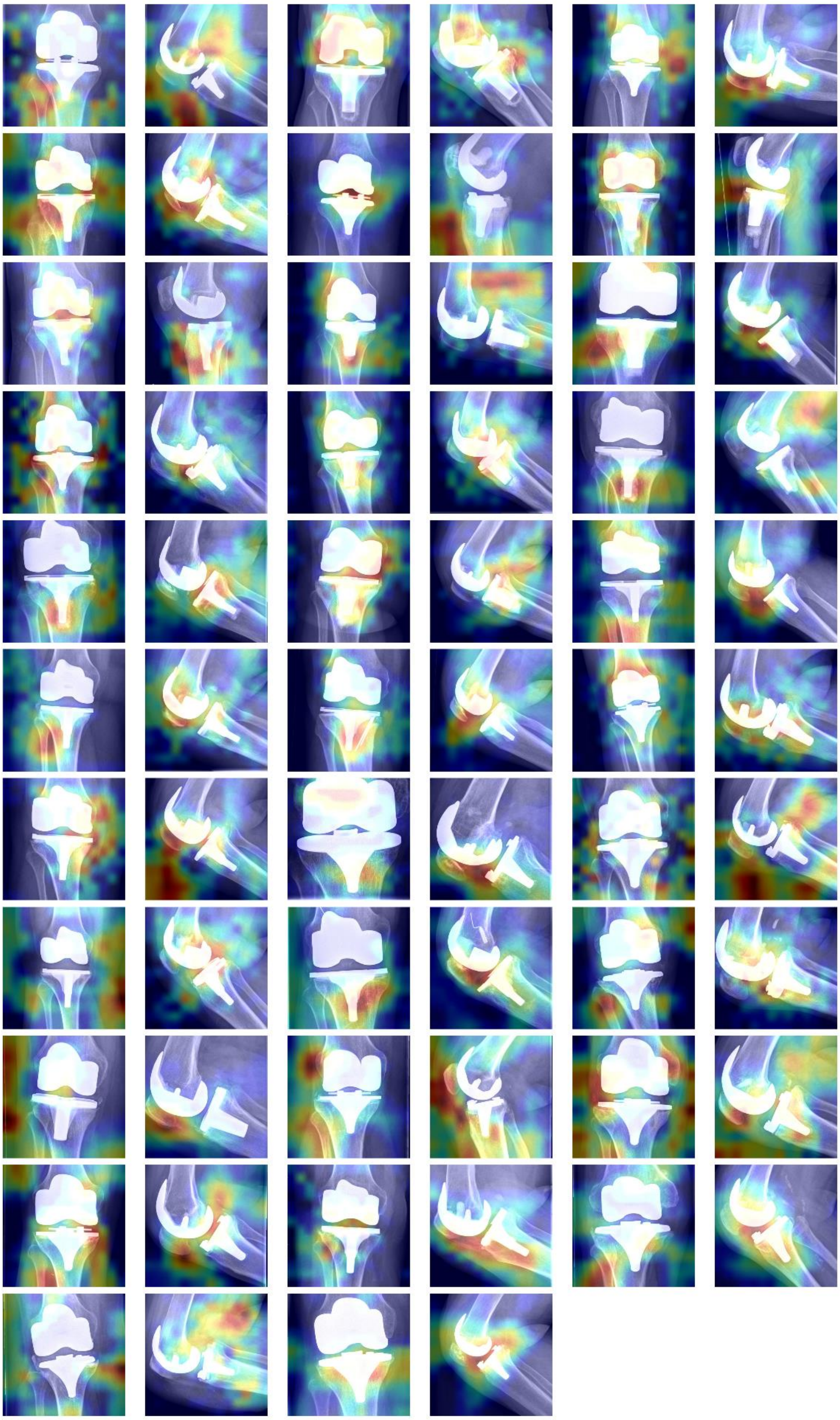

Figure 10: Gradient-weighted Class Activation Mapping (Grad-CAM) for all loosening cases in fold 5. Each panel shows the original anteroposterior and lateral radiographs alongside the corresponding Grad-CAM overlay.
